# Physical activity and overweight/obesity among Malaysian adults: findings from the 2015 National Health and morbidity survey (NHMS)

**DOI:** 10.1186/s12889-017-4772-z

**Published:** 2017-09-21

**Authors:** Ying Ying Chan, Kuang Kuay Lim, Kuang Hock Lim, Chien Huey Teh, Chee Cheong Kee, Siew Man Cheong, Yi Yi Khoo, Azli Baharudin, Miaw Yn Ling, Mohd Azahadi Omar, Noor Ani Ahmad

**Affiliations:** 1 Institute for Public Health, Ministry of Health Malaysia, Jalan Bangsar, 50590 Kuala Lumpur, Malaysia; 2 Institute for Medical Research, Ministry of Health Malaysia, Jalan Pahang, Kuala Lumpur, 50588 Malaysia

**Keywords:** Physical activity, Overweight, Obesity, BMI, National Health and Morbidity Survey

## Abstract

**Background:**

Overweight and obesity are growing health problems both worldwide and in Malaysia due to such lifestyle changes as decreased physical activity (PA), increased sedentary behavior and unhealthy eating habits. This study examined the levels and patterns of PA among normal-weight and overweight/obese adults and to investigate the association between PA level and overweight/obesity in Malaysian adults.

**Methods:**

This study used data from the 2015 National Health and Morbidity Survey (NHMS), a nationwide cross-sectional survey that implemented a two-stage stratified random sampling design. Respondents aged 18 years and above (*n* = 17,261) were included in the analysis. The short version of International Physical Activity Questionnaire (IPAQ) was administered to assess the respondents’ PA levels. The respondents’ height and weight were objectively measured and body mass index (BMI) was calculated. The respondents were categorized according to BMI as either normal-weight (18.5–24.9 kg/m^2^) or overweight/obese (≥ 25 kg/m^2^). Descriptive and complex sample logistic regression analyses were employed as appropriate.

**Results:**

Overall, approximately 1 in 2 respondents (51.2%) were overweight/obese, even though the majority (69.0%) reporting at least a moderate level of PA (total PA ≥ 10 MET-hours/week). In both normal-weight and overweight/obese groups, a significantly higher prevalence of high PA (total PA ≥ 50 MET-hours/week) was observed among men than women (*p* < 0.001), but women reported a significantly higher prevalence of low and moderate PA than men (*p* < 0.001). Men reported significantly higher activity levels (in MET-hours/week) than women with regard to walking, vigorous-intensity PA and total PA (*p* < 0.001). Overweight/obese men reported a significantly lower level of vigorous-intensity PA and total PA than normal-weight men (*p* < 0.001). A low level of PA was associated with the risk of overweight/obesity (Adjusted OR = 1.14; 95% CI: 1.01–1.30) compared to a high level of PA among men but not among women.

**Conclusions:**

The levels of PA were inversely related to the risk of overweight/obesity in men but not in women. Programs designed to reduce overweight/obesity rates should encourage the practice of moderate- to vigorous-intensity PA. Future research should consider using longitudinal and prospective approaches that simultaneously measure dietary intake, PA and BMI among Malaysian adults to investigate the actual relationship between PA and overweight/obesity.

**Electronic supplementary material:**

The online version of this article (doi:10.1186/s12889-017-4772-z) contains supplementary material, which is available to authorized users.

## Background

Overweight and obesity are growing public health problems that have become global epidemics [1]. Numerous reports have shown that various non-communicable diseases such as cardiovascular disease, type II diabetes mellitus, hypertension, dyslipidemia and certain types of cancer were related to overweight/obesity, which can further enhance the burden of diseases and the mortality rate [1–3]. Globally, the number of overweight and obese people increased almost three folds during the last three decades (from 857 million in 1980 to 2.1 billion in 2013), with proportion of females outweighed males [4]. In addition, worldwide in 2014, World Health Organization (WHO) reported that adults aged 18 years and older who were overweight and obese were 39% and 13%, respectively [5]. Overweight and obesity were once considered to only affect high-income countries, but they have increased tremendously in developing countries, predominantly among urban dwellers [5]. Social, economic and nutritional transitions coupled with reduced physical activity following rapid urbanization and modernization in these countries have influenced the health of their populations and communities.

Physical activity (PA) is defined as any bodily movement produced by skeletal muscles that results in energy expenditure [6]. Regular and adequate levels of PA in adults are key contributors to energy expenditure and are essential for energy balance and weight control [7]. Numerous studies have reported the importance of PA for weight control [8–10]. Furthermore, PA has been shown to reduce the risk of cardiovascular disease and other chronic diseases, including diabetes mellitus, hypertension, obesity, cancer (colon and breast), and osteoporosis [11]. According to the WHO guidelines (2010), an adult aged 18 to 64 years should perform at least 150 min/week of moderate-intensity aerobic PA, or 75 min/week of vigorous-intensity aerobic PA, or an equivalent combination of moderate- and vigorous-intensity PA, which is equivalent to a total PA level of at least 600 metabolic equivalent-minutes per week (MET-minutes/week) or 10 MET-hours/week [12]. Various types, amounts and intensities of PA are needed for different health outcomes. For instance, a person who engaged in regular PA of moderate-intensity for a duration of 30 min or more on most days of the week has a lower risk for cardiovascular disease, diabetes, colon cancer and breast cancer. However, for body weight control and to prevent unhealthy weight gain in adulthood, approximately 60 min of moderate- to vigorous-intensity PA in a day preferably all days per week may be needed [13]. An appropriate daily caloric intake is also recommended. Formerly overweight/obese people require approximately 60 to 90 min of moderate-intensity PA each day to maintain weight loss [14].

The prevalence of obesity has reached epidemic levels in many developing countries, and Malaysia is of no exception. Based on previous National Health and Morbidity Surveys (NHMSs) carried out in 2006, 2011 and 2015, an increasing trend of overweight and obesity prevalence was observed among Malaysian adults aged 18 years and older: 29.1% (95% CI: 28.6–29.7) and 14.5% (95% CI: 13.6–14.5) in 2006 [15], 29.4% (95% CI: 28.4–30.4) and 15.1% (95% CI: 14.3–15.9) in 2011 [16], 30.0% (95% CI: 29.1–31.0) and 17.7% (95% CI: 16.9–18.5) in 2015, respectively [17]. According to a systematic analysis of global data on the prevalence of overweight and obesity in adults [4], the prevalence of obesity in Malaysia (11.4% in males; 16.7% in females) was observed to be lower than that reported in Western countries, such as Australia (27.5% in males; 29.8% in females) and the United States (31.7% in males; 33.9% in females), but almost three to four times higher than in other Asian countries, such as India (3.7% in males; 4.2% in females), China (3.8% in males; 5.0% in females), Taiwan (4.3% in males; 6.4% in females) and Japan (4.5% in males; 3.3% in females).

Overweight and obesity have been linked to various factors, including physical inactivity, unhealthy dietary habits, alcohol intake, socioeconomic conditions and genetic factors [18]. Changes in individual lifestyle behaviors, such as a lack of PA and increased sedentary behavior associated with rapid urbanization, may lead to an increasing prevalence of overweight and obesity. However, studies among Malaysian populations have yielded conflicting results with an increased prevalence of overweight and obesity, despite increasing rates of PA participation. Statistics from the NHMS show that Malaysian adults aged 18 years and older are becoming more physically active, with the prevalence of PA (achieving at least 10 MET-hours/week of total PA) increasing from 56.3% (95% CI: 55.5–57.1) in 2006 [19] to 64.8% (95% CI: 63.6–66.1) in 2011 [16] and 66.9% (95% CI: 65.8–68.0) in 2015 [17]. Compared to other countries that have used the IPAQ, the prevalence of PA in Malaysia is slightly higher than in Taiwan (57.7%) and Japan (56.7%) but considerably lower than in China (93.1%) and Australia (82.9%) [20]. Despite increasing PA, Malaysia is now ranked as Southeast Asia’s fattest country according to recent reports by the WHO, with the percentages of overweight or obese men and women being 43.8% and 48.6%, respectively [4].

Based on a previous national nutrition survey conducted in Malaysia, Malaysian adults had a daily median energy intake of 1466 kcal/day or 64% of the Malaysian Recommended Nutrient Intakes (RNI), with men (1489 kcal/day) reporting a higher median energy intake than women (1445 kcal/day) [21]. The study observed that energy intake among the Malaysian population falls below the recommended intake level. However, the study had a relatively small sample size and nearly half of the studied subjects had under-reported their energy intakes [21]. The study may not reflect the true energy intake of the Malaysian population and cautious interpretation of the findings is required. The national nutrition survey was conducted as a 10-year survey in Malaysia. In 2015, the energy intake of the Malaysian population was not measured because it was not included in the scope of the 2015 NHMS. The national nutrition surveys should be conducted regularly at shorter intervals to provide information on the dietary trends and energy intake patterns of the Malaysian population.

As the 2015 NHMS reported that a substantial proportion of Malaysian adult population were overweight and obese and a high prevalence of PA [17], the question of why an increasing number of Malaysians are overweight or obese, even with increasing PA participation, subsequently arises. Are Malaysians sufficiently physically active to counter the rise in overweight and obesity? Despite extensive evidence on the beneficial effect of PA on body weight [8–10], studies on the association between PA levels and overweight and obesity among the Malaysian population are lacking. Therefore, the aims of this study were to examine PA levels (low, moderate, and high PA) and patterns of PA (walking, moderate-intensity PA, vigorous-intensity PA and total PA) in normal-weight and overweight/obese adults and to determine the associations between PA levels and overweight/obesity among Malaysian adults. This study could generate important evidence to support policy-making decisions in designing effective PA programs and interventions for overweight and obese populations.

## Methods

### Study design and sampling

This study was based on data on adults aged 18 years and older from the 2015 NHMS, a cross-sectional survey with a two-stage proportionate to size cluster sampling design to ensure national representativeness. The 2015 NHMS was a nationwide household survey that targeted all non-institutionalized Malaysian residents. The stratification was performed by states and urban/rural localities. States and Federal Territories constituted the Primary Stratum, and urban and rural areas within the states were considered the Secondary Stratum. The sampling process was completed by the Department of Statistics Malaysia using an updated 2014 sampling frame. Based on the frame, Malaysia was divided into contiguous geographical areas called ‘enumeration blocks’ (EBs). We included all states and Federal Territories in this survey. Within each state, selected numbers of EBs from urban and rural areas were randomly selected. Each EB consisted of 80 to 120 living quarters (LQs) with an average population of 500 to 600 people. Using the probability-proportional-to-size sampling technique, a total of 869 EBs (536 urban and 333 rural) were randomly chosen from the total EBs in Malaysia (approximately 75,000). In each selected EB, a total of 12 LQs were randomly chosen, and all eligible household members within the selected LQs were invited to participate in this survey. A detailed methodology and sampling design of the survey is described in the NHMS 2015 official report [22].

### Data collection

Data were collected from March to June 2015 by trained evaluators via face-to-face interviews using a standardized pre-validated structured questionnaire. The questionnaire was programmed into an application, known as the e-NHMS 2015 application, and uploaded onto Samsung Galaxy tablets as mobile data collection tools. The tablets were used to collect data, store and back up data in the SD cards, and upload data to the central system. A total of 19,767 eligible adults aged 18 years and older who were living in the selected households were invited to participate in this survey. Vacant or closed houses during the initial visit were revisited up to three times to maintain the required sample size. Information sheets and consent forms were given to all eligible respondents prior to the interviews. The information sheet and consent form were read aloud to respondents who are illiterate and signified by a thumb print. The Medical Research and Ethics Committee (MREC), Ministry of Health Malaysia granted the permission to carry out the study (NMRR-14-1064-21,877).

### Physical activity assessment

The short version of International Physical Activity Questionnaire (IPAQ) was used to measure physical activity. The reliability and validity of the IPAQ have been proven in 12 different countries [23]. In this survey, we adopted the Malay version of the IPAQ, which was previously pilot-tested and pre-validated in our previous 2011 NHMS [24]. The short version IPAQ in both Malay language (Bahasa Malaysia) and English language was used to measure self-reported physical activity during the past 1 week. The physical activity level and intensity were calculated in metabolic equivalent task minutes per week (MET-minutes/week) based on the IPAQ scoring protocol [25]. Total minutes spent on vigorous activity, moderate-intensity activity, and walking over the last 7 days were multiplied by 8.0, 4.0, and 3.3, respectively, to compute MET scores for each activity. The total physical activity score was calculated as the sum of all MET scores from the three sub-components. Physical activity was categorized into low, moderate and high PA level according to the scoring rules of the short version IPAQ [25]. In the present study, PA in MET-minutes/week was transformed and presented as MET-hours/week to enhance the consistency and interpretability of our findings.

### Sedentary behavior

Sedentary behavior was measured as part of the short version IPAQ based on the IPAQ sitting question but was not included in any part of the derived physical activity summary score. Respondents were asked to think about the total time that they spent (hours per day) sitting or lying down, including in the workplace, in the house, in their free time and while travelling by vehicle, excluding time spent sleeping, on a typical day. Total daily sitting time was used as a proxy of overall sedentary behavior. The respondents were categorized into: (i) those who have at least 8 h of total sitting time per day and (ii) those who have less than 8 h of total sitting time per day. This categorization was applied according to evidence from previous studies which reported total sitting time of more than 8 h a day is positively associated with all-cause mortality [26].

### Anthropometric measurements

The height and weight of the respondents (wearing only light clothing and bare-footed) were measured using the SECA Portable Stadiometer 213 (SECA GmbH & Co. KG, Hamburg, Germany) and the TANITA Digital Weighing Scale HD 319 (TANITA Corp., Tokyo, Japan) to the nearest 0.1 cm and 0.1 kg, respectively. The average value of two measurements was used for data entry to minimize measurement error. Bedridden subjects, amputees or those with limbs in plaster, pregnant women and those who had given birth within less than 60 days prior to the interview were excluded from the anthropometric measurements. Body mass index (BMI) was calculated as weight divided by height squared (kg/m^2^). In this study, we categorized the respondents into two BMI groups, normal-weight (18.5–24.9 kg/m^2^) and overweight/obese (≥ 25.0 kg/m^2^), based on the WHO cutoff points for BMI [1]. All analyses were performed based on these two groups. We excluded underweight (BMI < 18.5) respondents in the analysis because our study focuses on the comparison between overweight/obese and normal-weight groups.

### Control variables

In this study, several socio-demographic variables and cardiometabolic health status were included as control variables in the regression model. Age (categorized into 18–29 years, 30–39 years, 40–49 years, 50–59 years, and 60 years and above), ethnic groups (Malays, Chinese, Indian, other Bumiputeras, and “Others”), residential area (urban and rural), marital status (single, married, widow/widower/divorcee), educational level (no formal education, primary, secondary and tertiary education) and monthly household income [in Malaysian Ringgit (MYR) (1 USD ≈ 4.44 MYR); grouped into less than MYR1000, MYR1000-MYR1999, MYR2000-MYR2999, MYR3000-MYR3999, MYR4000-MYR4999, and MYR5000 and above] were considered potential covariates in the sex-specific analysis.

The presence or absence of cardiometabolic comorbidities, such as diabetes, hypertension and hypercholesterolemia, was assessed according to respondents’ self-reported information on these health conditions as diagnosed by a doctor or healthcare professional. The respondents were asked to answer “yes” or “no” to the question “Have you ever been told by a doctor or Assistant Medical Officer that you have diabetes, hypertension or hypercholesterolemia?”

### Statistical analyses

Descriptive statistic was used to illustrate the socio-demographic and other characteristics of the respondents according to BMI status. Pearson’s Chi-square test for categorical variables was used to analyze differences in characteristics between the study variables and BMI status. The prevalence of “low PA”, “moderate PA” and “high PA” by BMI status among men and women was examined. Statistically significant differences between men and women in each BMI group were tested using Chi-square tests. Mean values of physical activity (MET-hours/week) and sedentary behavior (hours/day) were also examined relative to BMI status among men and women. Statistical comparisons between men and women in each BMI group were performed using the independent t-test. Statistically significant differences between normal-weight and overweight/obese groups within sexes were also tested. Univariate and multivariate logistic regression analyses were performed separately for men and women to investigate the associations between physical activity level (low, moderate, or high) and overweight/obesity with adjustments for other covariates. Crude and adjusted odds ratios (ORs) with 95% confidence intervals (CIs) were calculated. Selected variables (with a *p*-value of <0.25 in the univariate analysis) were included in the final multivariate analysis model [27]. The multivariate analysis was performed for men and women separately to examine the independent association between PA level and overweight/obesity while adjusting for all other potential covariates such as socio-demographic characteristics, sedentary behavior, and cardiometabolic comorbidities. The statistical significance level was set at 0.05. All statistical analyses were done using SPSS version 22.0 (IBM Corp., Armonk, NY, USA). Sample weights and study design were taken into consideration using a complex sampling design in all data analyses. The extended Mantel-Haenszel chi-square for linear trend analysis was performed using OpenEpi Version 3.01, an open source software program for epidemiologic statistics, to investigate whether there were any dose-response relationships between physical activity level (low, moderate, high) and the risk of overweight/obesity among Malaysian adults aged 18 years and older.

## Results

Out of 19,767 eligible adults, 18,296 respondents completed the PA assessment and anthropometric measurements in the 2015 NHMS giving a response rate of 92.6% in this study. To focus on the comparison between overweight/obese and normal-weight groups, we excluded underweight respondents (*n* = 1035). Therefore, the final sample included 17,261 respondents.

Table 1 shows the characteristics of the respondents by BMI status. We combined overweight and obese adults into one group. Of the 17,261 respondents, approximately half were overweight/obese (51.2%). The majority of the respondents were Malays (49.0%), urban dwellers (76.0%), married (66.6%), and had a secondary level education (46.9%). Regarding physical activity level, most of the respondents reported moderate PA (42.2%) followed by low PA (31.0%) and high PA (26.8%). Most of the respondents (85.9%) reported less than 8 h of sitting time per day. A small proportion of respondents reported having diabetes (8.6%), hypertension (13.8%), and hypercholesterolemia (9.7%). The percentage of overweight/obese individuals increased with age and level of education. A bigger proportion of overweight/obese individuals were made up of Indians (65.3%) compared to other ethnic groups. Respondents who reported low or moderate PA constituted a higher proportion of overweight/obese individuals compared to those who reported high PA. Respondents with diabetes, hypertension or hypercholesterolemia also represented a high percentage of overweight/obese individuals.

**Table 1:** Characteristics of respondents by BMI status, NHMS 2015

Characteristic		BMI status		
	Total sample	Normal-weight	Overweight/Obese	*p*-value (Chi-square)
	n (%)	n (%)	n (%)	
Overall	17,261 (100.0)	7718 (48.8)	9543 (51.2)	
Sex				0.003
Men	8287 (52.6)	3984 (50.3)	4303 (49.7)	
Women	8974 (47.4)	3734 (47.1)	5240 (52.9)	
Age group (years)				< 0.001
18–29	3900 (30.5)	2265 (60.0)	1635 (40.0)	
30–39	3480 (23.6)	1510 (48.0)	1970 (52.0)	
40–49	3374 (18.5)	1255 (40.3)	2119 (59.7)	
50–59	3315 (14.7)	1199 (38.3)	2116 (61.7)	
≥ 60	3192 (12.7)	1489 (47.7)	1703 (52.3)	
Ethnicity				< 0.001
Malays	10,678 (49.0)	4411 (44.1)	6267 (55.9)	
Chinese	2676 (22.5)	1455 (56.5)	1221 (43.5)	
Indians	1238 (7.0)	452 (34.7)	786 (65.3)	
Other Bumiputeras	1562 (11.1)	727 (47.5)	835 (52.5)	
Others	1107 (10.4)	673 (65.0)	434 (35.0)	
Residential area				0.067
Urban	9976 (76.0)	4473 (48.2)	5503 (51.8)	
Rural	7285 (24.0)	3245 (50.7)	4040 (49.3)	
Marital status				< 0.001
Single	3402 (26.5)	1973 (59.1)	1429 (40.9)	
Married	12,211 (66.6)	5025 (44.9)	7186 (55.1)	
Widow/widower/divorcee	1648 (6.9)	720 (46.3)	928 (53.7)	
Educational level				0.002
No formal education	1110 (5.6)	583 (55.4)	527 (44.6)	
Primary	4226 (21.0)	1876 (49.2)	2350 (50.8)	
Secondary	7927 (46.9)	3423 (47.0)	4504 (53.0)	
Tertiary	3806 (26.4)	1730 (49.7)	2076 (50.3)	
Monthly household income (1 USD = 4.44 MYR)				0.824
< MYR1000	2721 (13.2)	1263 (50.5)	1458 (49.5)	
MYR1000-MYR1999	3137 (16.4)	1423 (49.4)	1714 (50.6)	
MYR2000-MYR2999	2934 (16.4)	1318 (48.5)	1616 (51.5)	
MYR3000-MYR3999	2217 (13.0)	964 (48.0)	1253 (52.0)	
MYR4000-MYR4999	1535 (9.7)	691 (48.5)	844 (51.5)	
≥ MYR5000	4717 (31.3)	2059 (48.3)	2658 (51.7)	
Physical activity level				<0.001
Low	5230 (31.0)	2371 (49.0)	2859 (51.0)	
Moderate	7221 (42.2)	3080 (46.6)	4141 (53.4)	
High	4751 (26.8)	2233 (51.7)	2518 (48.3)	
Sedentary behaviour				0.584
Yes (≥ 8 h sitting time/day)	2197 (14.1)	992 (48.0)	1205 (52.0)	
No (< 8 h sitting time/day)	14,986 (85.9)	6683 (48.8)	8303 (51.2)	
Diabetes				< 0.001
Yes	1869 (8.6)	558 (30.0)	1311 (70.0)	
No	15,392 (91.4)	7160 (50.5)	8232 (49.5)	
Hypertension				< 0.001
Yes	2908 (13.8)	815 (28.8)	2093 (71.2)	
No	14,353 (86.2)	6903 (52.0)	7450 (48.0)	
Hypercholesterolemia				< 0.001
Yes	2016 (9.7)	593 (31.3)	1423 (68.7)	
No	15,245 (90.3)	7125 (50.6)	8120 (49.4)	

Table 2 shows the prevalence of low, moderate and high PA by BMI status among men and women. For both BMI groups (normal-weight and overweight/obese), a significantly higher prevalence of high PA was observed among men than women (*p* < 0.001), but the prevalence of low and moderate PA was significantly higher among women than men (*p* < 0.001). Regardless of BMI status, the prevalence of high PA was approximately twice as high in men as in women (Table 2). Among men, the overweight/obese group reported a significantly lower prevalence of high PA compared to the normal-weight group (*p* < 0.001). Among women, the overweight/obese group reported a slightly higher prevalence of moderate PA and high PA compared to the normal-weight group (*p* = 0.021) (see Additional file 1: Table S1).

**Table 2:** Prevalence of low, moderate and high PA levels by BMI status among men and women, NHMS 2015

Variable	BMI status					
	Normal-weight			Overweight/Obese		
	Men	Women	*p*-value^a^	Men	Women	*p*-value^a^
PA level, % (95% CI)			< 0.001			< 0.001
Low	26.5 (24.6–28.5)	36.8 (34.7–39.0)		28.4 (26.4–30.3)	33.4 (31.5–35.3)	
Moderate	35.2 (33.3–37.2)	46.4 (44.3–48.6)		39.5 (37.6–41.5)	48.6 (46.7–50.5)	
High	38.3 (35.9–40.7)	16.7 (15.2–18.4)		32.1 (30.2–34.1)	18.0 (16.6–19.6)	

^a^ *p*-value for the differences between men and women in each BMI group using Chi-square test

Table 3 shows the mean values of physical activity (MET-hours/week) and sedentary behavior (hours of sitting time/day) for men and women according to BMI status. In both BMI groups, men reported significantly higher activity levels (in MET-hours/week) than women with regard to walking, vigorous-intensity PA and total PA (*p* < 0.001). Among men, total PA and vigorous-intensity PA levels were significantly lower in the overweight/obese group compared to the normal-weight group (*p* < 0.001) (see Additional file 2: Table S2). Among women, the overweight/obese group reported a slightly higher level of moderate-intensity PA compared to the normal-weight group (*p* = 0.028). There were no significant differences observed for activity levels of walking, vigorous-intensity PA or total PA between normal-weight women and overweight/obese women (see Additional file 2: Table S2). In terms of sedentary behavior, no significant differences were observed across the mean hours of sitting time/day between BMI groups or between men and women (Table 3).

**Table 3:** Mean values of physical activity (MET-hours/week) and sedentary behaviour (hours/day) for men and women according to BMI status, NHMS 2015

Variable	BMI status					
	Normal-weight			Overweight/Obese		
	Men	Women	*p*-value^a^	Men	Women	*p*-value^a^
Physical activity, MET-hours/week (95%CI)						
Walking	8.7 (7.8–9.6)	6.5 (5.8–7.2)	< 0.001	8.5 (7.7–9.2)	6.4 (5.9–6.9)	< 0.001
Moderate-intensity PA	13.9 (12.5–15.2)	14.4 (13.3–15.4)	0.768	12.9 (11.7–14.1)	16.0 (14.9–17.0)	< 0.001
Vigorous-intensity PA	48.4 (42.5–54.3)	10.1 (8.4–11.8)	< 0.001	34.4 (31.5–37.4)***	9.6 (8.4–10.8)	< 0.001
Total PA	70.8 (64.0–77.6)	30.9 (28.7–33.2)	< 0.001	55.1 (51.7–58.5)***	31.9 (30.0–33.8)	< 0.001
Sedentary behaviour, hours/day (95% CI)	4.0 (3.9–4.2)	4.2 (4.1–4.4)	0.133	4.1 (4.0–4.3)	4.1 (3.9–4.3)	0.122

^a^ *p*-value for the differences between men and women in each BMI group using t-test

***p< 0.001, compared with normal weight group, within sex

Table 4 shows the logistic regression analyses for overweight/obesity among men and women. Multivariate analysis showed that respondents (only men, not women) with a lower level of PA (moderate PA level) were more likely to be overweight/obese (OR = 1.14; 95% CI: 1.01–1.30) compared to those with a high level of PA. Relative to a high level of PA, a low level of PA showed no significant association with the risk of overweight/obesity in this study. No significant association was observed between sedentary behavior and overweight or obesity among men and women. Among the socio-demographic variables, age, ethnicity and educational level were significantly associated with being overweight and obese in both sexes. Regarding marital status, married men (OR = 1.38; 95% CI: 1.15–1.67) were more likely to be overweight or obese than single men. The odds of overweight and obesity were high among those with hypertension and male adults with diabetes and hypercholesterolemia.

**Table 4:** Univariate and multivariate logistic regression analyses for overweight/obesity among Malaysian adults aged 18 years and above, NHMS 2015

Characteristic	Sex			
	Men		Women	
	Crude OR (95% CI)	Adjusted OR^a^ (95% CI)	Crude OR (95% CI)	Adjusted OR^a^ (95% CI)
Physical activity level				
Low	1.28 (1.10–1.49)**	1.06 (0.91–1.24)	0.84 (0.72–0.98)*	0.93 (0.79–1.09)
Moderate	1.34 (1.18–1.51)***	1.14 (1.01–1.30)*	0.97 (0.85–1.11)	1.02 (0.87–1.18)
High	1.00	1.00	1.00	1.00
Age group (years)				
18–29	1.00	1.00	1.00	1.00
30–39	1.31 (1.10–1.57)**	1.14 (0.93–1.40)	2.10 (1.77–2.49)***	2.10 (1.73–2.55)***
40–49	1.98 (1.65–2.37)***	1.44 (1.14–1.81)**	2.55 (2.15–3.01)***	2.33 (1.91–2.83)***
50–59	1.96 (1.63–2.35)***	1.24 (0.98–1.58)	3.06 (2.61–3.59)***	2.48 (2.01–3.06)***
≥ 60	1.32 (1.10–1.58)**	0.74 (0.56–0.98)*	2.09 (1.75–2.49)***	1.67 (1.31–2.19)***
Ethnicity				
Malays	1.14 (0.98–1.33)	1.23 (1.04–1.47)*	2.50 (2.13–2.94)***	2.87 (2.40–3.43)***
Chinese	1.00	1.00	1.00	1.00
Indians	1.59 (1.18–2.13)**	1.54 (1.13–2.10)**	4.00 (3.12–5.13)***	4.18 (3.19–5.47)***
Other Bumiputeras	0.99 (0.78–1.25)	1.17 (0.91–1.50)	2.20 (1.75–2.78)***	2.57 (1.97–3.37)***
Others	0.44 (0.34–0.57)***	0.56 (0.41–0.75)***	1.31 (0.98–1.74)	1.51 (1.10–2.08)*
Residential area				
Urban	1.41 (1.21–1.63)***	1.15 (1.00–1.33)	0.84 (0.74–0.96)**	1.02 (0.88–1.17)
Rural	1.00	1.00	1.00	1.00
Marital status				
Single	1.00	1.00	1.00	1.00
Married	1.64 (1.43–1.89)***	1.38 (1.15–1.67)**	1.95 (1.69–2.25)***	1.02 (0.86–1.22)
Widow/widower/divorcee	1.23 (0.86–1.76)	1.24 (0.83–1.86)	1.88 (1.56–2.28)***	0.86 (0.67–1.10)
Educational level				
No formal education	1.00	1.00	1.00	1.00
Primary	1.56 (1.14–2.13)**	1.21 (0.88–1.66)	1.29 (1.04–1.60)*	1.35 (1.07–1.71)*
Secondary	2.15 (1.58–2.92)***	1.50 (1.11–2.04)**	1.09 (0.89–1.34)	1.26 (0.99–1.62)
Tertiary	2.49 (1.81–3.44)***	1.67 (1.20–2.33)**	0.74 (0.60–0.93)**	1.01 (0.76–1.34)
Monthly household income (1 USD = 4.44 MYR)				
< MYR1000	1.00	1.00	1.00	1.00
MYR1000-MYR1999	1.07 (0.86–1.33)	1.13 (0.89–1.44)	1.10 (0.90–1.33)	1.12 (0.92–1.37)
MYR2000-MYR2999	1.14 (0.93–1.41)	1.07 (0.84–1.35)	1.11 (0.92–1.34)	1.13 (0.93–1.37)
MYR3000-MYR3999	1.26 (1.00–1.60)*	1.15 (0.89–1.49)	1.03 (0.85–1.26)	1.10 (0.89–1.36)
MYR4000-MYR4999	1.31 (1.01–1.71)*	1.10 (0.83–1.46)	0.94 (0.78–1.18)	1.05 (0.82–1.35)
≥ MYR5000	1.42 (1.16–1.74)**	1.22 (0.97–1.53)	0.87 (0.73–1.05)	1.10 (0.90–1.34)
Sedentary behaviour				
Yes (≥ 8 h sitting time/day)	1.22 (1.02–1.46)*	1.15 (0.96–1.38)	0.86 (0.74–1.01)	0.94 (0.80–1.10)
No (< 8 h sitting time/day)	1.00	1.00	1.00	1.00
Diabetes				
Yes	2.44 (2.00–2.98)***	1.34 (1.05–1.72)*	2.31 (1.91–2.79)***	1.21 (0.98–1.49)
No	1.00	1.00	1.00	1.00
Hypertension				
Yes	2.76 (2.31–3.29)***	2.16 (1.75–2.68)***	2.58 (2.20–3.02)***	2.08 (1.74–2.49)***
No	1.00	1.00	1.00	1.00
Hypercholesterolemia				
Yes	2.73 (2.21–3.38)***	1.58 (1.25–1.99)***	1.87 (1.58–2.20)***	1.12 (0.93–1.35)
No	1.00	1.00	1.00	1.00

^a^Odds ratios (ORs) adjusted for all other variables shown in the table

Statistically significant. *p< 0.05; **p< 0.01; ***p< 0.001

The extended Mantel-Haenszel chi-square test for both sexes showed that an increased level of physical activity was significantly associated with a lower risk of overweight and obesity (a dose-response relationship) after adjusting for age (*p* = < 0.001). When the test was performed for each sex separately and stratified by age, the associated *p*-values for men and women were *p* = < 0.001 and *p* = 0.048, respectively, suggesting that a dose-response relationship exists in both men and women (data not shown).

## Discussion

Our study explored differences in the levels and patterns of PA between normal-weight and overweight/obese adults and examined the relationship between PA level and overweight/obesity among a representative sample of Malaysian adults. We observed that slightly more than half of Malaysian adults (51.2%) were overweight or obese (BMI ≥ 25), although nearly 70% of subjects reported at least a moderate level of PA (total PA ≥ 10 MET-hours/week). This finding means that most of the participants met the minimum level of 600 MET-minutes/week (10 MET-hours/week) of total PA as recommended by the WHO (2010) [12] but have an overweight or obese BMI. An individual may need to do more than the minimum recommended level of PA to lose weight [28]. However, the exact amount and intensity of PA needed to maintain or lose weight is not clear, since these requirements vary between different individuals and change according to the energy intake of a person [28]. Our study revealed that overweight or obese adults were less likely to engage in vigorous-intensity PA compared to normal-weight adults and that this phenomenon is only observed among men (not among women). The levels of PA were observed to be inversely associated with the risk of overweight/obesity in men but not in women.

In this study, regardless of BMI status, men reported a twice higher prevalence of high PA compared to women, while the prevalence of low and moderate PA was significantly higher among women than men. Men also reported higher activity levels than women with regard to vigorous-intensity PA and total PA as reported in previous studies [29, 30]. This finding may be explained by greater participation in vigorous-intensity activities, such as sports and exercise, among men and a higher level of light- and moderate-intensity activities, such as household chores among women. We observed that men in the overweight/obese group engaged in less vigorous-intensity PA and total PA compared to men in the normal-weight group. Similar results have been reported in other studies [31, 32]. One possible explanation is that it may be more difficult for overweight/obese men to perform vigorous-intensity PA (e.g., jogging, running, swimming, and hiking) due to their poor physical condition. However, whether less physical activity causes overweight and obesity or vice versa is unclear. Among women, the level of PA and PA intensity did not differ significantly between the normal-weight and overweight/obese groups. Women have been identified as the least active subgroup in our previous studies [24], and the low level of PA among women may contribute to the non-significant findings of this study.

After adjusting for potential confounders, we observed that the practice of moderate-level PA was associated with a greater likelihood of overweight/obesity compared to high-level PA among men only. No significant association was observed between PA levels and overweight/obesity among women in this study. Similarly, a study conducted among Mexican adults also observed that a high PA level was inversely associated with overweight/obesity among men, but not among women [33]. This finding may be attributed to the higher prevalence of high PA levels noted in men compared to women as high PA tends to have a greater effect in reducing obesity. Moderate PA may not be sufficient to lower BMI [34]. In addition, the non-significant association between PA levels and BMI among women may be explained by the possible overestimation of self-reported physical activity in overweight/obese women [33]. The finding that men who reported a moderate PA level had a higher risk of being overweight or obese according to the logistic regression may be related to excess energy intake and/or reduced energy expenditure from insufficient physical activity intensity. A higher intake of energy (food) can suppress the beneficial effects of physical activity on weight [35]. Low PA alone does not necessarily contribute to a higher BMI because weight gain only occurs when there is an increase in energy intake coupled with reduced energy expenditure [35]. However, data regarding regular food intake to evaluate energy intake among the Malaysian population was not available in this study.

Regarding sitting time and BMI status, self-reported total sitting time in hours/day was not significantly different between normal-weight and overweight/obese groups. In contrast to the significant association between PA levels and overweight/obesity found in men, no significant association was observed between sitting time and overweight/obesity for either men or women in this study. It should be noted that sedentary behavior (sitting time) and self-reported PA are not linked and are not determined by the same factors [36, 37]. In other words, being sedentary is not the same as being insufficiently physically active. An individual can be simultaneously highly active and highly sedentary (e.g. a person who has a desk job that requires sitting for 9 h a day, but later jogs or runs every day after work). Additionally, measurement errors of the amount of sedentary time or the confounding effects of food intake might be another plausible reason for the non-significant association sedentary behavior and overweight/obesity among men and women in our study. Previous studies have shown mixed findings regarding the association between sitting time and BMI. A Portuguese study among 4091 Azorean men revealed a positive association between total sitting time (hours/day) and BMI after adjustments for all potential confounders including total PA [38]. Another study by Wanner et al. (2016) revealed a positive relationship between sitting time and percent body fat, but not BMI [39]. The authors suggested that BMI may not be the best measure of body weight as it could not differentiate between fat and muscle mass [38]. It is also noteworthy that the assessment of sitting the by IPAQ short form is limited to only one question without further division by domains [25].

The influence of physical activity on overweight/obesity is complex and likely bidirectional. Physical activity has been commonly recommended for weight control. However, a recent review by Cook and Schoeller (2011) [40] concluded that supporting evidence for physical activity for weight control is still inconclusive. It is crucial to understand that obesity is a complex problem of energy balance and a result of complex behavioral, psychological, environmental, and/or genetic factors. Different intensities of physical activity may have different effects on overweight and obesity. A previous study by Tan et al. (2015) [34] among 2380 Malaysian adults aged 25–64 years suggested that a high level of PA or vigorous-intensity PA is needed for healthy changes in body weight. In our study, we observed that overweight/obese men were less likely to practice vigorous-intensity PA compared to normal-weight men. Our hypothesis that a lower level of PA is associated with a higher risk of overweight/obesity is only true in men (not in women). However, to our knowledge, no previous study has evaluated the association between PA and overweight/obesity in a representative nationwide sample of Malaysian adult population of both sexes. Our findings might contribute to the formulation or modification of current policies and suggest that effective PA programs designed for overweight and obese populations should emphasize vigorous-intensity PA, rather than light- or moderate-intensity PA.

Our findings also suggest that several socio-demographic factors (i.e., age, ethnicity, marital status and educational level) and cardiometabolic comorbidities, such as diabetes, hypertension and hypercholesterolemia, were associated with overweight and obesity. Similar to findings reported in a previous national study in Malaysia [41], Indians were associated with the highest risk of overweight/obesity among the different ethnic groups. Genetic predispositions to overweight or obesity among Indians and other environmental factors, including behavioral and cultural influences on food preparation and consumption, could increase their susceptibility to obesity [42]. Among men, the upper middle-age group (40 to 49 years old) was more likely to be overweight/obese, and the likelihood decreased in the elderly (≥ 60 years old) compared to their younger (18 to 29 years old) counterparts. This finding is similar to findings in most other studies [43–45]. Mean body weight and BMI tend to decrease as both lean body mass and fat mass decrease after the age of 60 years [46]. For women, increasing age is positively associated with overweight/obesity as reported in previous studies [47–49]. Women of childbearing age tend to gain the most weight [50]. In addition, rapid hormone changes during the menopausal transition may also lead to weight gain and increased central fat distribution in middle-aged and older women [51]. Men with higher educational levels were more likely to be overweight or obese. This finding was consistent with a Korean study in which a significant positive association between education and obesity was observed in men [52]. This finding may be attributed to the tendency of better-educated men to obtain better jobs or more sedentary occupations, leading to physical inactivity during working days, and live more modern and sedentary lifestyles. Therefore, specific attention and public health intervention strategies should target subgroups (middle-aged adults, Indians, individuals who are married and have a higher educational level) that are at a greater risk of overweight and obesity. Previous approaches such as healthy lifestyle programs and the ‘10,000 steps a day’ campaign implemented by the Malaysian government need to be reinforced to combat overweight and obesity. The electronic and printed mass media may serve as the most powerful channel to reach different levels of society to educate and raise awareness on healthy body weight management [53]. The Malaysian government has implemented strong initiatives to reduce the obesity rate. However, long-term, persistent and focused efforts are required to counteract the rising rates of overweight and obesity.

This study was not without limitations. First, the causal relationship between physical activity and BMI status cannot be established by the cross-sectional study design. Second, the energy intake of the study population was not measured. Therefore, no matching can be done between energy intake and energy expenditure to determine the energy balance and body weight of an individual. Further longitudinal and prospective studies that measure food intake, physical activity, and weight changes simultaneously are warranted to examine the actual relationship between physical activity and overweight/obesity. Third, the IPAQ is less accurate compared with objective measurements of physical activity, such as pedometers and accelerometers. Respondents’ self-report data on physical activity may be subject to a potential recall bias, resulting in under-reporting of actual behavior. Responses may also be more susceptible to a social desirability bias (e.g., over-reporting of physical activity), particularly in face-to-face interviews. Lastly, other variables such as dietary intake, body fat and smoking were not included in the current study.

This study also has several strengths. First, the NHMS is a large-scale population-based survey with a large sample size that enables generalization of the results to the Malaysian adult population. Second, objectively measured anthropometric data are more accurate than respondents’ self-report anthropometric data. Third, face-to-face interviews by trained evaluators and the high response rate enhance the validity of the collected data. Furthermore, the use of the validated short version IPAQ in our study enables comparisons with other study populations locally and internationally.

## Conclusions

Our findings provide additional information on the association between PA and BMI and suggest that a lower level of PA is associated with a higher risk of overweight/obesity. Although the association was only observed in men, actions taken to reduce overweight and obesity should involve both men and women. Future studies on the association between PA and BMI should consider using longitudinal and prospective approaches that simultaneously measure dietary intake, PA and BMI among the Malaysian adult population to provide more concrete evidence for policy-makers pertaining to the relationship between PA and BMI. Interventions and strategies aimed at preventing and controlling overweight/obesity should encourage the practice of moderate- to vigorous-intensity PA and promote healthy eating. Such interventions should be feasible and should specifically target overweight and obese populations who are at a high risk of chronic diseases. Long-term efforts and commitments from all stakeholders are required to combat the obesity epidemic and its associated cardiometabolic comorbidities.

## Additional files

Additional file 1: Table S1. The prevalence of low, moderate and high PA levels by BMI status among men and women from the 2015 NHMS. These data show the prevalence of low, moderate and high PA levels between normal-weight and overweight/obese individuals. The data were analyzed for men and women separately. (DOC 35 kb)

Additional file 2: Table S2. Mean values of physical activity (MET-hours/week) for men and women according to BMI status from the 2015 NHMS. These data show the mean MET-hours/week of physical activity between normal-weight and overweight/obese individuals. The data were analyzed for men and women separately. (DOC 60 kb)

## Abbreviations

BMI: Body mass index
CI: Confidence interval
IPAQ: International physical activity questionnaire
MET: Metabolic equivalent
NHMS: National health and morbidity survey
OR: Odds ratio
PA: Physical activity
WHO: World Health Organization
