# Supplementary material for: Physical activity and overweight/obesity among Malaysian adults: findings from the 2015 National Health and morbidity survey (NHMS)
Source: BMC Public Health. 2017 Sep 21;17:733. doi: 10.1186/s12889-017-4772-z (PMC5609047; doi:10.1186/s12889-017-4772-z)
Supplement: Supplementary file 1 — The prevalence of low, moderate and high PA levels by BMI status among men and women from the 2015 NHMS. These data show the prevalence of low, moderate and high PA levels between normal-weight and overweight/obese individuals. The data were analyzed for men and women separately. (DOC 35 kb) [file 12889_2017_4772_MOESM1_ESM.doc]

Table S1: Prevalence of low, moderate and high PA levels by BMI status among men and women, NHMS 2015

| **Variable** | **Men** | |  |  | **Women** | |  |
| --- | --- | --- | --- | --- | --- | --- | --- |
|  | **Normal-weight** | **Overweight/Obese** | ***p*-value^a^** |  | **Normal-weight** | **Overweight/Obese** | ***p*-value^a^** |
| PA level, % (95%CI) |  |  | <0.001 |  |  |  | 0.021 |
| Low | 26.5 (24.6-28.5) | 28.4 (26.4-30.3) |  |  | 36.8 (34.7-39.0) | 33.4 (31.5-35.3) |  |
| Moderate | 35.2 (33.3-37.2) | 39.5 (37.6-41.5) |  |  | 46.4 (44.3-48.6) | 48.6 (46.7-50.5) |  |
| High | 38.3 (35.9-40.7) | 32.1 (30.2-34.1) |  |  | 16.7 (15.2-18.4) | 18.0 (16.6-19.6) |  |
|  |  |  |  |  |  |  |  |

^a^*p*-value for the differences between normal-weight and overweight/obese groups in each sex using Chi-square test.
