# Supplementary material for: Physical activity and overweight/obesity among Malaysian adults: findings from the 2015 National Health and morbidity survey (NHMS)
Source: BMC Public Health. 2017 Sep 21;17:733. doi: 10.1186/s12889-017-4772-z (PMC5609047; doi:10.1186/s12889-017-4772-z)
Supplement: Supplementary file 2 — Mean values of physical activity (MET-hours/week) for men and women according to BMI status from the 2015 NHMS. These data show the mean MET-hours/week of physical activity between normal-weight and overweight/obese individuals. The data were analyzed for men and women separately. (DOC 60 kb) [file 12889_2017_4772_MOESM2_ESM.doc]

Table S2: Mean values of physical activity (MET-hours/week) for men and women according to BMI status, NHMS 2015

| **Variable** | **Men** | |  |  | **Women** | |  |
| --- | --- | --- | --- | --- | --- | --- | --- |
|  | **Normal-weight** | **Overweight/Obese** | ***p*-value^a^** |  | **Normal-weight** | **Overweight/Obese** | ***p*-value^a^** |
| Physical activity,  MET-hours/week (95%CI) |  |  |  |  |  |  |  |
| Walking | 8.7 (7.8-9.6) | 8.5 (7.7-9.2) | 0.394 |  | 6.5 (5.8-7.2) | 6.4 (5.9-6.9) | 0.367 |
| Moderate-intensity PA | 13.9 (12.5-15.2) | 12.9 (11.7-14.1) | 0.026 |  | 14.4 (13.3-15.4) | 16.0 (14.9-17.0) | 0.028 |
| Vigorous-intensity PA | 48.4 (42.5-54.3) | 34.4 (31.5-37.4) | <0.001 |  | 10.1 (8.4-11.8) | 9.6 (8.4-10.8) | 0.167 |
| Total PA | 70.8 (64.0-77.6) | 55.1 (51.7-58.5) | <0.001 |  | 30.9 (28.7-33.2) | 31.9 (30.0-33.8) | 0.656 |
|  |  |  |  |  |  |  |  |

^a^*p*-value for the differences between normal-weight and overweight/obese groups in each sex using t-test.
